# Fever in childbirth: a mini-review of epidural-related maternal fever

**DOI:** 10.3389/fnins.2024.1389132

**Published:** 2024-04-19

**Authors:** Yuki Kinishi, Yoshihisa Koyama, Tomoo Yuba, Yuji Fujino, Shoichi Shimada

**Affiliations:** ^1^Department of Anesthesiology and Intensive Care Medicine, Osaka University Graduate School of Medicine, Suita, Japan; ^2^Department of Neuroscience and Cell Biology, Osaka University Graduate School of Medicine, Osaka, Japan; ^3^Addiction Research Unit, Osaka Psychiatric Research Center, Osaka Psychiatric Medical Center, Osaka, Japan; ^4^Global Center for Medical Engineering and Informatics, Osaka University, Suita, Japan; ^5^Integrated Frontier Research for Medical Science Division, Institute for Open and Transdisciplinary Research Initiatives (OTRI), Osaka University, Suita, Japan

**Keywords:** epidural-related maternal fever, intrapartum fever, epidural anesthesia, interleukin-1 receptor antagonist, sterile inflammation

## Abstract

Fever during childbirth, which is often observed in clinical settings, is characterized by a temperature of 38°C or higher, and can occur due to infectious and non-infectious causes. A significant proportion of non-infectious causes are associated with epidural-related maternal fever during vaginal delivery. Therapeutic interventions are required because fever has adverse effects on both mother and newborn. Effective treatment options for ERMF are lacking. As it is difficult to distinguish it from intrauterine infections such as chorioamnionitis, antibiotic administration remains the only viable option. We mentioned the importance of interleukin-1 receptor antagonist in the sterile inflammatory fever pathway and the hormonal influence on temperature regulation during childbirth, an important factor in elucidating the pathophysiology of ERMF. This review spotlighted the etiology and management of ERMF, underscoring recent advancements in our understanding of hypothalamic involvement in thermoregulation and its link to *sterile inflammation*. We propose to deepen the understanding of ERMF within the broader context of autonomic neuroscience, aiming to foster the development of targeted therapies.

## 1 Introduction

The incidence of maternal fever during childbirth ranges from 1.6 to 14.6% among all delivering women (Burgess et al., 2017). There are two primary causes of maternal fever: infectious and non-infectious. Regardless of the etiology, fever negatively affects both the mother and newborn, necessitating therapeutic intervention. Notably, a significant proportion of non-infectious causes are associated with epidural-related maternal fever (ERMF) during vaginal delivery; however, there is currently no effective treatment available for ERMF. As it is difficult to distinguish it from intrauterine infections, such as chorioamnionitis, antibiotic administration remains the only viable option.

In contrast to the content covered in the cited work (Patel et al., 2023), this mini review emphasized recent advances in understanding the role of the hypothalamus as a thermoregulatory center, particularly regarding its involvement in the sterile inflammatory processes underlying ERMF. Moreover, we highlighted novel insights into the sterile inflammatory fever pathway, exploring the influence of hormonal changes occurring during childbirth and their implications for thermoregulation in late pregnancy, aspects crucial for unraveling the pathophysiology of ERMF. This review aims to integrate the latest information and propose new research directions, thereby providing a fresh perspective on ERMF and its management within the broader context of autonomic neuroscience.

## 2 Maternal fever in childbirth

### 2.1 Definition of maternal fever

Generally, a body temperature above 38°C is defined as fever requiring intervention (Goetzl, 2023). Oral temperature is considered an accurate reflection of intrauterine temperature (Banerjee et al., 2004). Importantly, maternal oral temperature and fetal core temperature differ by 1.6°C: When the maternal temperature is 38°C, the fetal core temperature is 39.6°C; when the maternal temperature is 39°C, the fetal core temperature is 40°C or higher.

### 2.2 Etiology of maternal fever

Maternal fever during delivery involves infectious and/or non-infectious etiologies and differs between preterm and full-term deliveries (Goetzl, 2023). Fever associated with preterm labor is typically infectious, whereas fever in full-term labor may be attributed to intrauterine infections, including chorioamnionitis, or non-infectious etiologies, such as ERMF. Reports indicate chorioamnionitis presents in 3–5% of deliveries (Kim et al., 2015), and 20% of pregnant women who receive epidural anesthesia develop ERMF (Sultan et al., 2016). Less common causes of noninfectious fever include prostaglandin E2 use, dehydration, hypothyroidism, and increased ambient temperature (Goetzl, 2023).

### 2.3 Maternal and fetal outcomes related to intrapartum fever

Regardless of its etiology, fever affects both the mother and newborn. Maternal effects include decreased uterine contractions, increased cesarean delivery rates, and increased postpartum hemorrhage (Goetzl, 2023). Meanwhile, neonatal adverse outcomes include conditions such as neonatal encephalopathy, respiratory distress syndrome, meconium aspiration syndrome, neonatal intensive care unit admission, fetal acidosis, and low Apgar scores. The likelihood of neonatal encephalopathy is further increased by exposure to risk factors, such as acidosis, hypoxemia, infection, and inflammation (Sultan and Segal, 2020). The odds ratios for these adverse outcomes increase with higher fever levels (Dior et al., 2016; Hensel et al., 2022).

## 3 Clinical and basic studies on pathophysiology of ERMF

### 3.1 Clinical features in ERMF

ERMF was first reported by Fusi et al. in 1989. Inflammatory cytokines such as IL-6 increase at the onset of labor (Neal et al., 2015). ERMF occurs primarily during vaginal delivery. During elective cesarean section, epidural anesthesia inhibits norepinephrine-induced vasoconstriction, increasing heat loss from the skin, and resulting in hypothermia (Horn et al., 2002). In non-pregnant surgical populations, fever associated with epidural anesthesia is not observed because it is suppressed by inhaled anesthetics and opioids (Kurz et al., 1995; Negishi et al., 1998). As fever associated with epidural anesthesia has not been reported in many clinical situations other than labor, the underlying mechanism of ERMF may be related to the unique circumstances of labor. However, despite various studies on the risk factors for ERMF, recent results from a systematic review have not identified any independent or causative factors associated with ERMF (Chang et al., 2023).

### 3.2 Understanding ERMF: thermoregulation and labor epidural anesthesia—proposed mechanism of ERMF

The hypothalamus, a thermoregulatory center, receives information from various temperature receptors in the body, provides continuous feedback on internal and external temperature changes, and organizes autonomic and behavioral responses, including shivering, sweating, and controlling vasoconstriction, to maintain deep temperatures within an optimal range. The main effect of epidural anesthesia is sympathetic blockade by the suppression of nerve impulses. The cutaneous vascular tone is regulated by the noradrenergic vasoconstrictor and cholinergic vasodilator pathways (Kellogg, 2006). Although cutaneous vascular tone is regulated by noradrenergic vasoconstriction under normothermic and hypothermic conditions, cholinergic vasodilation is responsible for up to 80% of the increase in cutaneous blood flow under hyperthermic conditions (Kellogg, 2006). In nonpregnant patients and pregnant women undergoing elective cesarean section, epidural anesthesia inhibits vasoconstriction and increases cutaneous heat loss, decreasing body temperature (Matsukawa et al., 1995; Horn et al., 2002).

The pathophysiology of ERMF has not been fully elucidated; however, two hypotheses have been proposed: (1) sterile inflammation induced by local anesthetics and (2) thermoregulation with epidural anesthesia (Patel et al., 2023). Clinical research suggests that steroid administration prevents the development of ERMF (Goetzl et al., 2006), supporting the sterile inflammation hypothesis. This suggests that ERMF is a pathogen-free (noninfectious) placental inflammation (Riley et al., 2011). *In vitro* studies have demonstrated that steroids suppress the inflammatory signals induced by ropivacaine in human umbilical vein endothelial cells and placental trophoblasts (Wohlrab et al., 2020). In addition, bupivacaine-induced reduction in caspase-1 activity impairs the release of the anti-inflammatory cytokine IL-1ra in human leukocytes (Del Arroyo et al., 2019). These findings suggest that local anesthetics induce immune cell and mitochondrial dysfunction and increase inflammatory cytokines through inflammasome activation and that IL-1ra may play an important role in the pathophysiology of sterile inflammation (Patel et al., 2023). Conversely, the second hypothesis, alteration of thermoregulation with epidural anesthesia, involves several factors, including suppression of sweating (Fusi et al., 1989), thermoregulatory vasoconstriction (Kellogg, 2006), baroreceptor-mediated reflex vasoconstriction (a physiological response to a decrease in mean blood pressure; Baron et al., 1988), non-thermoregulatory vasoconstriction (increase in set point during fever; Stitt, 1979), suppression of cutaneous vasodilation (Kellogg, 2006), decreased heat release due to improved ventilation by analgesia (Hägerdal et al., 1983), and a decreased shivering threshold (Sessler, 2008; Segal, 2010). During an elective cesarean section, epidural anesthesia suppresses cutaneous vasoconstriction, resulting in a decreased body temperature (Horn et al., 2002). In contrast, cutaneous vasodilation induced by increased heat production is suppressed during vaginal delivery, resulting in an increased body temperature (Hägerdal et al., 1983; Segal, 2010). However, low concentrations of local anesthetics administered during labor are insufficient to explain the etiology of fever caused by epidural thermoregulation alone. Therefore, extent to which these two hypotheses contribute to ERMF development remains unclear. During labor, there is a physiological state of inflammation. It is reasonable to assume that epidural anesthesia increases the inflammatory response, causing heat production to exceed heat loss during labor, resulting in an increase in body temperature.

What is the main source of inflammation induced by local anesthetics? It remains unclear whether peripheral tissues, such as the placenta, are systemically inflamed, or the inflammation involves immune cells in the blood, or both. To the best of our knowledge, there are few animal studies on ERMF. In particular, mechanisms in the brain, such as changes in cytokine expression in the hypothalamus, are not well understood.

### 3.3 Unraveling the mechanisms: fever in sterile inflammation

Basic studies on the thermogenic pathway often use animal models of bacterial infection, such as those in which lipopolysaccharide is administered, and the cyclooxygenase (COX)-2-dependent pathway is well known (Blomqvist and Engblom, 2018). COX-2 activation produces prostaglandin E_2_ (PGE_2_), which causes fever via an autonomic mechanism driven by binding to EP3 receptors expressed on thermoregulatory neurons in the preoptic area (POA). Binding of PGE_2_ and EP3 receptor decreases the activity of descending inhibitory neurons in the POA and de-represses excitatory sympathetic pathways from the dorsomedial hypothalamus (DMH) and rostral globus pallidus through the medullary raphe nucleus, signaling increased thermogenesis. Through the release of norepinephrine, heat production in brown adipose tissue is increased, inducing vasoconstriction in the extremities, and reducing passive heat loss. Additionally, signaling through the neurotransmitter acetylcholine increases body temperature by stimulating the musculoskeletal system and causing shivering (Tansey and Johnson, 2015). Recent studies, including those by Mota et al. in 2022, have highlighted a COX-2-independent pathway in the IL-1β-induced fever response. The activation of glutamate receptors within the DMH plays an important role, although the precise input pathways to the DMH, whether the POA, other central neural circuits, or direct peripheral cytokine signals, remain to be fully elucidated (Mota and Madden, 2022). In addition, the anti-inflammatory effect of IL-1ra was demonstrated in a rat model of placental inflammation induced by administration of uric acid (Brien et al., 2021), a sterile inflammatory substance like IL-1β. Altogether these findings suggest the need for further studies of COX-2-independent pathways and mechanism of suppression of IL-1 system signaling by IL-1ra to elucidate the mechanisms of fever associated with sterile inflammation such as ERMF.

### 3.4 Ovarian hormones and the IL-1 system: their role in ERMF

**Figure 1:**
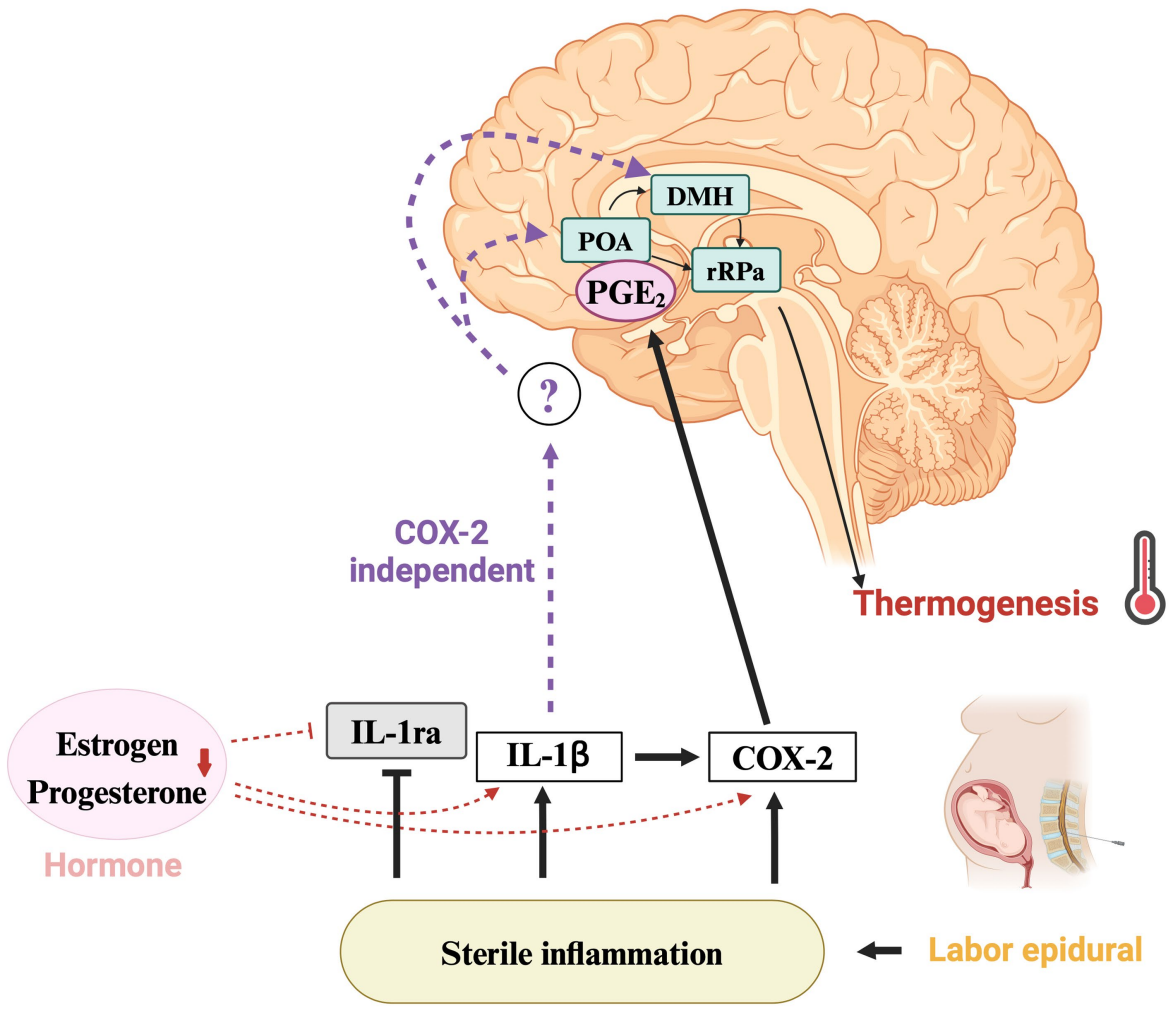
Mechanisms of fever regulation and the role of IL-1ra in ERMF. Thermoregulatory pathway implicated in epidural-related maternal fever (ERMF) during childbirth. COX-2-dependent and -independent pathways of sterile inflammation lead to fever via IL-1ra suppression. The decrease in hormones such as estrogen and progesterone contribute to lower IL-1ra levels and increase COX-2 expression in the hypothalamus, along with higher IL-1β plasma concentrations. The question mark highlights the yet-to-be-elucidated COX-2 independent pathway in the hypothalamus, suggesting a direction for further investigation. IL-1ra, interleukin-1 receptor antagonist; IL-1β, interleukin-1β; COX-2, cyclooxygenase-2; PGE_2_, prostaglandin E_2_; POA, preoptic area; DMH, dorsomedial hypothalamus; rRPa, rostral raphe pallidus. Created using BioRender.com.

The IL-1 system, which is important for immune and inflammatory responses, plays an important role in pregnancy, influencing implantation, placental development, and initiation of labor (Equils et al., 2020). As pregnancy progresses, maternal tissues become exposed to fetal demands and physical stress, which induces IL-1β. Whether an inflammatory response occurs may depend on the level of IL-1ra, which regulates the activity of IL-1β. During pregnancy and childbirth, thermoregulation is influenced by a complex interplay of immune response and inflammation. Fever is suppressed in late pregnancy through a variety of mechanisms, including changes in inflammatory cytokines such as IL-1, IL-6, and tumor necrosis factor-α and anti-inflammatory cytokines such as IL-1ra; decreased sensitivity to prostaglandins; and changes in ovarian hormones such as estrogen and progesterone (Mouihate et al., 2008). Ovarian hormones have been shown to suppress the febrile response, leading to decreased COX-2 expression in the hypothalamus and reduced plasma IL-1β levels (Mouihate and Pittman, 2003). In the luteal phase, ovarian hormones and IL-1ra levels have been shown to be positively correlated (Vetrano et al., 2020). However, the specific effects of changes in ovarian hormones on the febrile response and cytokine levels immediately before parturition are still unknown. The relationship between ovarian hormones and the fever pathway during sterile inflammation is summarized in Figure 1. Since ERMF occurs only during parturition, it is likely that IL-1ra is involved in its etiology. Taken together, understanding the interaction between ovarian hormones and the IL-1 system may help reveal the mechanism of ERMF.

### 3.5 Challenges in managing ERMF

The prophylactic administration of antibiotics and antipyretics is ineffective for ERMF (Goetzl et al., 2004; Sharma et al., 2014). In clinical settings, discerning ERMF from intrauterine infections such as chorioamnionitis, remains challenging. Therefore, antibiotic administration is recommended to manage maternal fevers (Goetzl, 2023). Although approximately 90% of pregnant women with ERMF show improvement within hours of delivery (Gonen et al., 2000), studies on the relationship between ERMF and long-term outcomes are limited (Li et al., 2022). While the current management of ERMF presents challenges, the potential role of IL-1ra in preventing ERMF represents an exciting frontier in maternal healthcare and will be the focus of the following discussion.

## 4 Discussion: can ERMF be prevented?—interventions targeting IL-1ra

Currently, no evidence supports prophylactic or therapeutic interventions for ERMF (Cartledge et al., 2022). Recent studies have focused on the anti-inflammatory cytokine IL-1ra. Decreased circulating IL-1ra levels are associated with perinatal inflammation and ERMF, both of which increase the rate of obstetric interventions (Ali et al., 2022). Elevated IL-1ra levels due to genetic mutations have been demonstrated to be associated with lower cesarean section rates (Ackland et al., 2022). The efficacy of cytokine therapy targeting IL-1ra has been demonstrated for the treatment of rheumatoid arthritis and other diseases (Arnold et al., 2022). IL-1ra is safely used in human pregnancy and does not significantly increase adverse outcomes (Brien et al., 2021). Whether targeted intervention for IL-1ra can be clinically applied to prevent the development of ERMF warrants further research on the mechanism of fever, not only at the peripheral level but also in the hypothalamus, the central thermoregulatory center. We look forward to further basic research to understand the complete picture of ERMF.

## Author contributions

YuK: Writing – review & editing, Writing – original draft. YoK: Writing – review & editing, Writing – original draft. TY: Writing – review & editing, Writing – original draft. YF: Writing – review & editing. SS: Writing – review & editing.
